# Prediction of Diabetic Sensorimotor Polyneuropathy Using Machine Learning Techniques

**DOI:** 10.3390/jcm10194576

**Published:** 2021-10-02

**Authors:** Dae Youp Shin, Bora Lee, Won Sang Yoo, Joo Won Park, Jung Keun Hyun

**Affiliations:** 1 Department of Rehabilitation Medicine, College of Medicine, Dankook University, Cheonan 31116, Korea; sindae90@dkuh.co.kr; 2 Deargen, Co., Ltd., Daejeon 34051, Korea; 2bora@deargen.me; 3 Department of Endocrinology and Metabolism, College of Medicine, Dankook University, Cheonan 31116, Korea; smff03@hanmail.net; 4 Department of Laboratory Medicine, College of Medicine, Dankook University, Cheonan 31116, Korea; joowon@dankook.ac.kr; 5 Department of Nanobiomedical Science & BK21 NBM Global Research Center for Regenerative Medicine, Dankook University, Cheonan 31116, Korea; 6 Institute of Tissue Regeneration Engineering (ITREN), Dankook University, Cheonan 31116, Korea

**Keywords:** machine learning, diabetes mellitus, diabetic sensorimotor polyneuropathy, random forest, prediction, electrophysiology

## Abstract

Diabetic sensorimotor polyneuropathy (DSPN) is a major complication in patients with diabetes mellitus (DM), and early detection or prediction of DSPN is important for preventing or managing neuropathic pain and foot ulcer. Our aim is to delineate whether machine learning techniques are more useful than traditional statistical methods for predicting DSPN in DM patients. Four hundred seventy DM patients were classified into four groups (normal, possible, probable, and confirmed) based on clinical and electrophysiological findings of suspected DSPN. Three ML methods, XGBoost (XGB), support vector machine (SVM), and random forest (RF), and their combinations were used for analysis. RF showed the best area under the receiver operator characteristic curve (AUC, 0.8250) for differentiating between two categories—criteria by clinical findings (normal, possible, and probable groups) and those by electrophysiological findings (confirmed group)—and the result was superior to that of linear regression analysis (AUC = 0.6620). Average values of serum glucose, International Federation of Clinical Chemistry (IFCC), HbA1c, and albumin levels were identified as the four most important predictors of DSPN. In conclusion, machine learning techniques, especially RF, can predict DSPN in DM patients effectively, and electrophysiological analysis is important for identifying DSPN.

## 1. Introduction

Type 2 diabetes mellitus (T2DM), the most common form of diabetes, is a major disease in humans worldwide [1], and its incidence is increasing with aging and lifestyle changes [2]. There is evidence that half of T2DM patients experience neurological disorders and a progressive disability of nerve fibers in the course of diabetes, and serious neurological symptoms lead to poor quality of life [3]. Diabetic sensorimotor polyneuropathy (DSPN) is a common neurological complication resulting from neuroinflammation, mitochondrial dysfunction, and apoptosis due to hyperglycemia, dyslipidemia, and altered insulin signaling, and leads to various symptoms and signs, including neuropathic pain, decreased sensation, and foot ulceration [4,5]. The management of DSPN is not limited to controlling hyperglycemia, and multidisciplinary programs, such as patient education, lifestyle modification, and physical activity, are required to control various physical and psychological symptoms and foot complications [6]. Therefore, early detection and prediction of DSPN is very important in DM patients.

The classification of DSPN has been defined in previous studies [7,8,9,10]. Typical DSPN is the most common form in DM patients and chronic, symmetrical, and length-dependent sensorimotor polyneuropathy [11]. Tesfaye et al. defined the minimal criteria for typical DSPN to estimate severity: possible, probable, confirmed, and subclinical based on clinical symptoms and signs and electrophysiology [7]. Numerous staging and scoring systems have been developed to assess the severity of DSPN; however, choosing the optimal scoring system is confusing because the results of previous studies are different regarding which system is effective [12,13,14]. Electrophysiological assessments, including nerve conduction studies (NCS), are important for diagnosing DSPN objectively [15,16]; however, special equipment is needed, and these assessments cannot be performed routinely for patients without clinical symptoms or signs because of the discomfort caused by electrical stimulation or needle insertion. Because the pathophysiology of diabetic neuropathy reveals a broad spectrum of axonal involvement and segmental demyelination, electrophysiological findings also indicate both axonal degeneration and demyelination [17]. Numerous predisposing factors for the development of DSPN have been found [18,19,20,21]. DSPN is significantly correlated with poor glucose control [18,19], longer duration of diabetes, poor metabolic management, smoking and the presence of cardiovascular disease, and DSPN severity is correlated with hypertension, dyslipidemia, microalbuminuria, alcohol consumption, and body mass index [20,21]. Most previous studies on the prediction of DSPN used various statistical methods. While traditional statistical methods draw only population inferences from clinical information, recently developed machine learning (ML) methods focus on developing predictive models from general-purpose learning algorithms [22]. Therefore, ML is considered to be a better way to predict DSPN in DM patients.

ML is a computationally broad and powerful data mining technique that can accommodate a large set of proposed variables as inputs to identify factors related to the results of interest [23], and ML develops algorithms that can learn patterns and decision rules, such as early detection, prediction and diagnosis, from data that are attributable to the medical field. Recent studies have used various ML techniques to predict complications, including retinopathy, nephropathy, foot ulceration and DSPN, in T2DM patients [24,25,26,27,28], and ML was effective for prediction of DSPN severity [24], 3-year complication developments [25], high-risk retinopathy, and numerous complications in nonadherent T2DM [27]. Haque et al. found that machine learning algorithms, especially random forest (RF), were effective in predicting DSPN severity based on the scoring system using Michigan Neuropathy Screening Instrumentation [29], which is not used widely, and that study assessed only type 1 diabetes mellitus (T1DM) patients.

The purpose of the current study was to delineate whether machine learning techniques are more useful than traditional statistical methods for predicting DSPN in type 2 DM patients, and whether the widely used classification for DSPN, which is based on clinical and electrophysiological findings, is amenable to the use of predictive models.

## 2. Materials and Methods

### 2.1. Subjects

**Figure 1:**
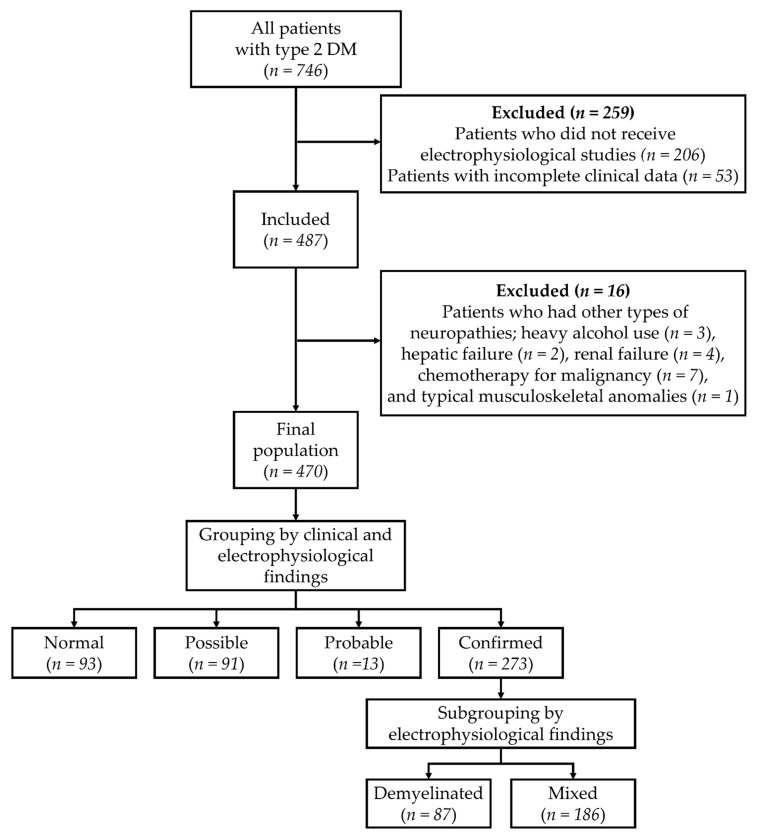
Flow and grouping of patients.

Medical records of patients with T2DM who visited Dankook University Hospital for the management of DM were collected, and 746 subjects were initially enrolled (Figure 1). Patients were diagnosed with T2DM by a physician at the Department of Endocrinology, based on the guideline of the American Diabetes Association [30]. Patients who did not undergo electrophysiological studies (*n* = 206) or had incomplete clinical data (*n* = 53) were excluded at first, and then patients who had other types of polyneuropathies, including heavy alcohol use (*n* = 3), hepatic failure (*n* = 2), renal failure (*n* = 4), chemotherapy for malignancy (*n* = 7), and typical musculoskeletal anomalies (*n* = 1), were subsequently excluded. As a result, 470 patients were included in the study (Figure 1). This study was approved by the Dankook University Hospital Institutional Review Board (IRB No. 2019-12-009).

### 2.2. Classification

Subjects were classified into 4 groups according to definitions of minimal criteria for typical DSPN based on the area of clinical care by Tesfaye et al. [7]: normal, possible, probable, and confirmed. The normal group (*n* = 93) consisted of subjects without any neurological symptoms or signs as previously described [7], and the possible group (*n* = 91) comprised subjects with one of the neurological symptoms or signs. The probable group (*n* = 13) comprised subjects with two or more neurological symptoms or signs. The confirmed group (*n* = 273) consisted of subjects with abnormal electrophysiological findings and neurological symptoms or signs. Electrophysiological assessments were performed according to the guidelines of the American Academy of Neurology [16], and NCS and electromyography of the upper and lower extremities were conducted. According to electrophysiological findings, the confirmed group was divided into two subgroups: A demyelinated subgroup (*n* = 87) with subjects who predominantly showed demyelination and a mixed subgroup (*n* = 186) with subjects who showed abnormal spontaneous activities during needle electromyography and demyelination (Figure 1).

### 2.3. Clinical Data

All subjects’ clinical information, such as baseline characteristics, past medical history, current health status, diabetic complications, and medications, was analyzed. Baseline characteristics included age, sex, weight, height, body mass index (BMI), disease duration (from initial diagnosis of T2DM to the date of the last follow-up at the hospital), smoking (current smoking, past smoking, or nonsmoking), family history of T2DM, and diabetes education. Past medical history included hypertension (HTN), dyslipidemia, and history of stroke and coronary artery disease. HTN was defined as systolic blood pressure > 140 mmHg, diastolic blood pressure > 90 mmHg or the use of antihypertensive medications. Diabetic retinopathy was included in diabetic complications. Medications for DM, HTN and dyslipidemia were included; medications for DM were metformin, sulfonylureas, thiazolidinediones (TZDs), dipeptidyl peptidase-4 inhibitors (DPP4is), sodium-glucose cotransporter-2 inhibitors (SGLT2is), and insulin; medications for HTN were calcium channel blockers (CCBs), angiotensin-converting-enzyme inhibitors (ACEis), angiotensin II receptor blockers (ARBs), beta blockers (BBs) and thiazides; and medications for dyslipidemia were statins. BMI was calculated as weight in kilograms divided by the square of height in meters.

### 2.4. Laboratory Data

**Figure 2:**
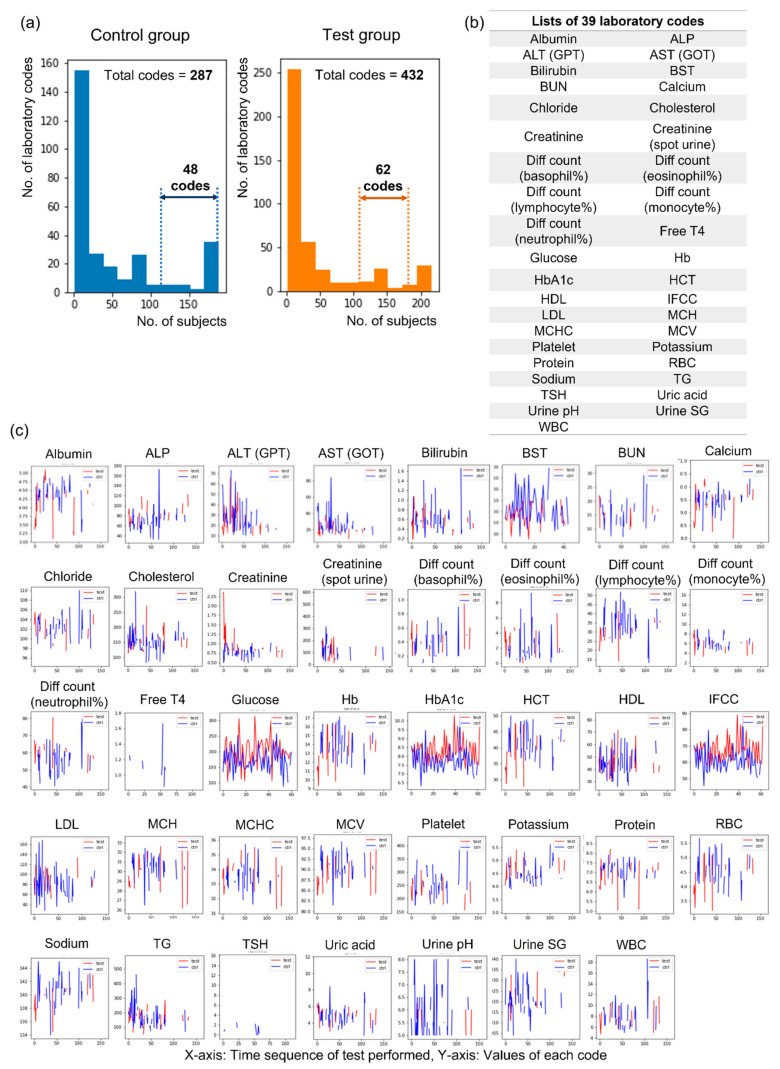
Selection of laboratory codes for machine learning analysis. (**a**) The distribution of laboratory codes according to tested subject numbers in the control and test groups, (**b**) lists of 39 selected laboratory codes, (**c**) graphs showing the changes in 39 selected laboratory codes at the initial and follow-up periods. Abbreviations: ALP = alkaline phosphatase; ALT (GPT) = alanine aminotransferase (glutamic pyruvate transaminase); AST (GOT) = aspartate aminotransferase (glutamic oxaloacetic transaminase); BST = blood sugar test; BUN = blood urea nitrogen; Diff = differential; T4 = thyroxine; Hb = hemoglobin; HbA1c = hemoglobin A1c; HCT = hematocrit; HDL = high-density lipoprotein cholesterol; IFCC = International Federation of Clinical Chemistry; LDL = low-density lipoprotein cholesterol; MCH = mean corpuscular hemoglobin; MCHC = mean corpuscular hemoglobin concentration; MCV = mean cell volume; PLT = platelet; RBC = red blood cell; TG = triglyceride; TSH = thyroid-stimulating hormone; SG = specific gravity; WBC = white blood cell.

A total of 432 laboratory codes from blood and urine tests were obtained from all subjects, and we divided subjects into a control group (*n* = 197) with normal electrophysiological findings and a test group (*n* = 273) with abnormal electrophysiological findings within the criteria of DSPN to identify the optimal number of laboratory codes (Figure 2). Forty-eight codes could be obtained for more than half of the subjects (*n* = 98) in the control group, and 62 codes could be obtained for more than half of the subjects (*n* = 135) in the test group (Figure 2a). When the results of the two groups were combined, 39 laboratory codes were ultimately selected (Figure 2b). Each laboratory code was assessed several times during the follow-up periods (range: 31–18368 days, mean value: 5202.9 days), and various changes in the values were observed within the period (Figure 2c).

Three methods were used to standardize the values of laboratory codes for ML analysis. Method 1 refers to the average value of each laboratory code during the follow-up period, method 2 is the first value of each laboratory code when T2DM was initially diagnosed while visiting the hospital, and method 3 refers to the pattern of laboratory code changes. The pattern was defined as −1, 0, and 1 as follows. If the initial value was 10% or more lower than the overall average of the values excluding the initial value, it was considered −1; if the change was less than 10%, it was regarded as 0; and if the initial value was greater than 10% of the overall average of the values excluding the initial value, it was regarded as 1.

### 2.5. Machine Learning Analysis

First, to define which variable set will be used for the classification model, a random forest (RF) model trained by different variable combinations was tested. As described above, there are four different variable sets: clinical data and methods 1, 2, and 3 for laboratory data. RF was trained with all possible combinations of four variable sets. Because of the limitation of the sample size, the sample was divided into ten groups, and each group was used as the test set. For each test set, the remainder of the samples were divided into a training set and a validation set at a 4:1 ratio by preserving the percentage of samples for each class. Fivefold cross-validation was performed for each test set, and the final performance was defined as the average of the performance over 10 iterations [31]. The combination set of clinical data and methods 1 and 3 for laboratory data (total, 105 variables) showed the best performance in cases of classifying patients [area under the curve (AUC) = 0.8350 and accuracy = 74.85%, Table 1; therefore, the combination set was used as an input variable for model training.

The DSPN predictor model was trained with the input variables identified above. The model performance was tested with the same method used when identifying the input variables. Three ML algorithms were used: XGBoost (XGB) [32], support vector machine (SVM) [33], and random forest (RF) [23], which were used alone or in combinations of two or more, that is, an ensemble of models for improvement of the model performance by fusion of the contents learned by different models and reduction of overfitting problems [34]. Among the various methods, the model averaging method for averaging the predicted values of several models was used in this work. AUC, accuracy, sensitivity, and specificity were used as performance metrics.

Finally, the feature importance of the best model among 7 models (XGB, SVM, RF, ensemble of XGB and SVM, ensemble of XGB & RF, ensemble of SVM and RF and ensemble of XGB and SVM and RF) was extracted from each model. If the best model was an ensemble of more than two models, the average feature importance obtained from each model was used as the feature importance of the ensemble model. Next, the models were retrained and evaluated with input features by adding features one by one, from the most to the least important. This was done to select the best set of features for DSPN prediction based on feature importance, and the performance was better when using the top 69 features for AUC and top 38 features for accuracy rather than all 105 features.

### 2.6. Statistics

To compare the predictability of ML results, traditional statistical methods were also carried out. All statistical analyses were performed with SPSS 26 (IBM, Armonk, NY, USA). The Shapiro-Wilk test was performed to assess the normal distribution of all quantified histological and functional data from each group. Categorical parameters were compared by likelihood ratio, and numerical parameters among groups were compared by one-way analysis of variance (ANOVA) and the Games–Howell post hoc test. Logistic regression was performed using statistically significant parameters and parameters that were identified to be important in previous studies, and the AUC, accuracy, sensitivity, and specificity were analyzed. *p*-values less than 0.05 were considered to indicate statistical significance.

## 3. Results

### 3.1. Baseline Characteristics among the Four Groups

When comparing baseline characteristics among the four groups, disease duration was significantly longer in the confirmed group than in the normal and possible groups (4543.18 ± 2849.75 days and 4464.03 ± 2934.87 days vs. 5686.67 ± 3648.57 days and in the normal, possible, and confirmed groups, respectively), and height was higher in the confirmed group than in the normal group (1.61 ± 0.09 m vs. 1.64 ± 0.09 m in the normal and confirmed groups, respectively). BMI and the initial values of BST and HbA1c were also different between the confirmed group and normal group and between the confirmed group and possible group (Table 2). The incidence of diabetic retinopathy was higher in the confirmed group (51.6%) than in the other groups (23.1–28.6%). Age; sex; weight; incidence of hypertension and dyslipidemia; smoking habit; past medical history of coronary artery disease, cerebrovascular disease, and stroke; and number of subjects who received diabetes education were not different among the groups (Table 2). Medications for diabetes control were different among groups; metformin (89.2–94.5%), sulfonylureas (68.1–68.8%), dipeptidyl peptidase-4 inhibitors (66.7–71.4%), and sodium-glucose cotransporter-2 inhibitors (17.2–20.9%) were used by a higher proportion of subjects in the normal and possible groups, whereas the proportion of subjects in the confirmed group who used insulin (65.6%) was higher than that in other groups (Table 2).

### 3.2. Identification of an Appropriate Classification for Prediction Using Machine Learning Analysis

Using ML algorithms, four groups of normal (A), possible (B), probable (C), and confirmed (D) samples were analyzed with various combinations. When comparing all groups separately (A vs. B vs. C vs. D) using the combined analysis of XGB and RF, the AUC was 0.8546, and the accuracy was 60.85% (Table 3). One of the classifications set to three groups (combination of A and B vs. C vs. D) showed the highest AUC value (0.8925) using the same analysis (XGB + RF); however, this classification was not appropriate because the number of group C patients was small (*n* = 13), which can result in imbalanced results [35]. When looking at the classification that combined group C with other groups, rather than alone, the classification with the combination of A, B and C vs. D showed a higher value of AUC (0.8250) than the other classifications and the highest value of accuracy (74.47%) (Table 3). Therefore, we performed all ML analyses and statistics based on this classification (A + B + C vs. D).

### 3.3. Identification of an Appropriate ML Algorithm for the Prediction of DSPN and Analysis of Predictive Values

**Figure 3:**
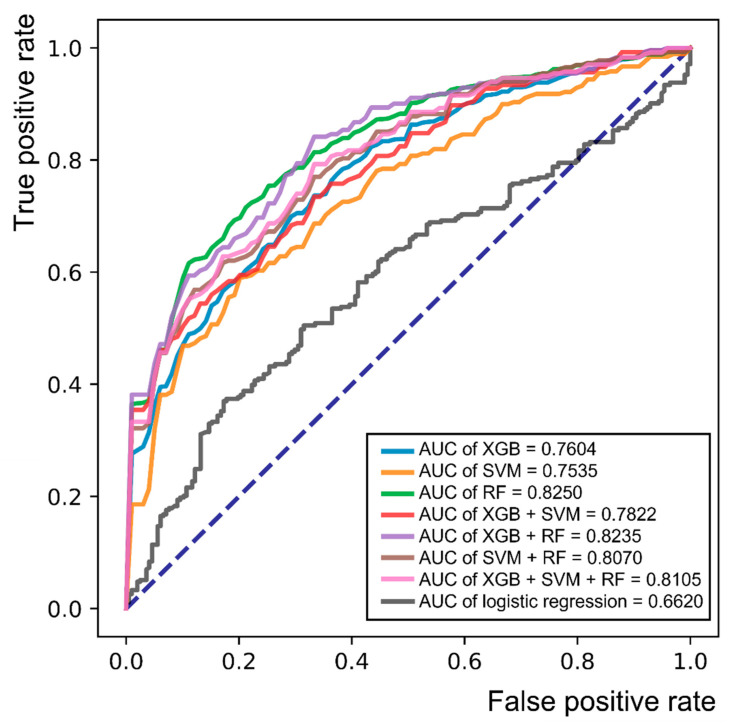
Receiver operating characteristic (ROC) curve for single or combinations of various machine learning algorithms and logistic regression analysis in the classification of the combination of the normal, possible, and probable groups versus the confirmed group. Abbreviations: AUC = area under the curve; XGB = XGBoost; RF = random forest; SVM = support vector machine.

When we compared various ML techniques (XGB, SVM, RF, and their combinations), RF showed the best AUC (0.8250) and accuracy (74.47%), and the sensitivity and specificity were also higher (0.7940 and 0.6720, respectively) than those of any other single algorithm or their combination (Table 4). Logistic regression analysis was performed to compare the combination of normal, possible, and probable groups with the confirmed group using meaningful parameters of the following basic characteristics and laboratory data: disease duration, initial value of HbA1c, DM retinopathy, family history of DM, use of metformin and insulin, serum levels of glucose, HDL cholesterol, albumin, and creatinine. The results of logistic regression analysis showed lower AUC (0.6620) and specificity (0.3519) values than RF. The receiver operating characteristic (ROC) curves of each ML algorithm and logistic regression analysis are shown in Figure 3. The AUC of RF was the highest (0.8250) among the 7 ML models, as described earlier, whereas the AUC of logistic regression was the lowest AUC value (0.6620).

### 3.4. Development of a Decision-Making Model Using Influential Features from the RF Algorithm

**Figure 4:**
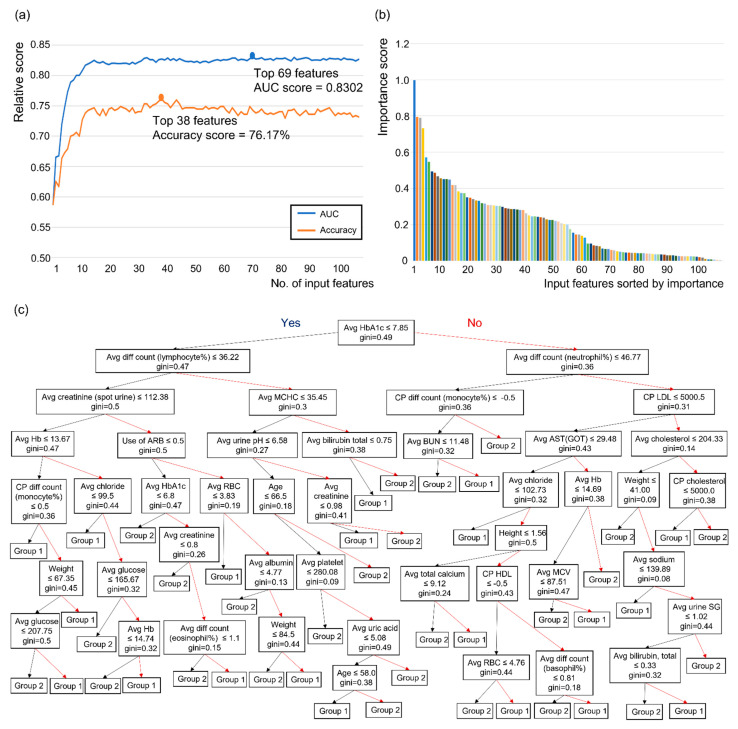
Application of random forest algorithm and process of extraction of important features in the classification of the combination of the normal, possible, and probable groups versus the confirmed group. (**a**) Model performance according to the number of input features sorted by importance, (**b**) the result of arranging input features in order of importance score, (**c**) a decision tree using the random forest algorithm with the classification of the combination of the normal, possible, and probable groups versus the confirmed group. Note: Group 1 = a group in which the normal, possible, and probable groups are combined, Group 2 = the confirmed group. Black arrow = positive results for the above features, red arrow = negative results for the above features, gini = gini index. Abbreviations: AUC = area under the curve; ALP = alkaline phosphatase; ALT (GPT) = alanine aminotransferase (glutamic pyruvate transaminase); AST (GOT) = aspartate aminotransferase (glutamic oxaloacetic transaminase); Avg = average; BST = blood sugar test; BUN = blood urea nitrogen; CP = change pattern; Diff = differential; T4 = thyroxine; Hb = hemoglobin; HbA1c = hemoglobin A1c; HCT = hematocrit; HDL = high-density lipoprotein cholesterol; IFCC = International Federation of Clinical Chemistry; LDL = low-density lipoprotein cholesterol; MCH = mean corpuscular hemoglobin; MCHC = mean corpuscular hemoglobin concentration; MCV = mean cell volume; PLT = platelet; RBC = red blood cell; TG = triglyceride; TSH = thyroid-stimulating hormone; SG = specific gravity; WBC = white blood cell.

RF analysis using the classification of the combination of the normal, possible, and probable groups versus the confirmed group was used to derive influential features, which consisted of clinical data and methods 1 and 3 for laboratory data. When these features are accumulated in the order of the importance score, the AUC and accuracy increase and then reach a maximum value at a certain moment (Figure 4a,b). In the case of AUC, the maximum value was reached when the number of parameters reached 69 (0.8302), and in the case of accuracy, the maximum value was reached when the number of parameters was 38 (76.17%) (Figure 4a,b). From this classification, the average value of HbA1c was identified as the first single discriminator for group determination between the combination of the normal, possible, and probable groups and the confirmed group (Figure 4c). The top 69 influential features are shown in Table 5. The average serum glucose level during the follow-up period was the most important feature (importance score = 0.997768) for determining the group in the classification, and the average values of the International Federation of Clinical Chemistry (IFCC; 0.794161), HbA1c (0.789265), and albumin levels (0.731579) during the follow-up period are shown in order of importance score (Table 5).

### 3.5. ML Analysis of the Confirmed Group to Identify Demyelinated and Mixed Types of DSPN

We compared the demyelinated subgroup with the mixed subgroup, as shown in electrophysiological studies of the confirmed group, using various ML algorithms and logistic regression analysis (Table 6). ML analysis revealed that the combination of XGB and SVM models showed the highest AUC and accuracy values of 0.5698 and 67.78%, respectively, whereas the statistical method using logistic regression showed a higher AUC value (0.6350). However, the overall AUC values of all ML algorithms and logistic regression analysis were much lower than the AUC value (0.8250) when RF was used to compare the combination of the normal, possible, and probable groups versus the confirmed group, and the specificity was quite low (0 and 0.3889 for RF and logistic regression, respectively) to predict the two subgroups within the confirmed group (Table 6).

## 4. Discussion

Interest in machine learning algorithms is widely increasing in the medical field because they can be used to predict disease development and generate semantic interpretations [36]. In the field of endocrinology, the prediction of diabetes is expected to be very useful for preventing disease progression and complications [37]. In this study, we have performed conventional statistics, as well as various ML algorithms to compare predictive power expressed in AUC and accuracy. Logistic regression analysis, a traditional statistical method, has an obvious limitation compared to the ML analysis. Only a small number of clinical and laboratory data (9 variables among over 400 data) were used during the statistical processing, which inevitably resulted in poor AUC whereas ML analysis could include over 100 meaningful data. Classical statistics usually draw population inferences, but become less precise when input variables that exceed the number of subjects, therefore appropriate ML method can help overcome this limitation [22].

As in all other fields, for the results of ML analysis to be more accurate, the input data must have extensive and accurate information. Laboratory data are usually obtained numerous times for a single subject during the follow-up period, and effective processing of meaningful data can have a significant impact on the establishment of predictive models. In this study, we tried various methods to optimize input data during the preprocessing step, especially for standardization of laboratory tests conducted at various time points. First, from the 432 types of laboratory data received for all patients, only 39 datapoints repeatedly obtained for more than half of all patients were filtered out. Then, depending on the timing of the laboratory data received, data were classified into average, initial, and change patterns of each value, and we found that average and changed patterns were meaningful parameters for ML analysis. Through these preprocesses, we are confident that we have increased the reliability of laboratory data and created a more accurate predictive model. When compared to previous studies that made predictive models of DSPN using ML algorithms in diabetic patients (Table 7), they did not explain what time point was used or whether there was any consideration of the amount of change in the laboratory data in addition to the data imputation process that handles missing data [24,25,27,38]. In addition, they did not provide any diagnostic tools, such as decision tree or nomogram, except Dagliati et al. [25].

Various criteria for defining DSPN have been developed, and many of them have been designed to classify the severity of DSPN based on clinical signs and symptoms alone [39] or in combination with physical examination [40,41] or electrophysiological findings [7,10]. Neurological signs, especially sensory abnormalities, are sensitive and specific findings for diagnosing DSPN and have been correlated with electrophysiological findings in previous studies [12,42,43]; however, we found that clinical data alone, which was categorized as normal, possible and probable groups defined in a previous study [7], was not effective in predicting DSPN in T2DM patients. Other studies have revealed that clinical symptoms and signs are too variable and inaccurate [44] and do not correlate well with the development of pathophysiological changes in the peripheral nervous system [13]. On the basis of our results, we confirmed that severity grading based on clinical symptoms and signs is not helpful and that electrophysiological assessment is essential in predicting DSPN. However, small fiber involvement, which is frequently occurs in early DSPN, is not identified by conventional NCS. Therefore, more specialized diagnostic tools such as quantitative sensory testing, skin biopsy, and corneal confocal microscopy are needed to identify small fiber damage [45,46].

We failed to classify the demyelinated and mixed types in the confirmed group in this study. Axonal involvement is frequently observed in DSPN, as is demyelination [17], and even axonal loss, which precedes demyelination, in sural nerves or plantar nerves of DSPN patients might be a primary finding [47,48]. Electrophysiological analysis, which shows decreased conduction velocity of sensory and motor nerves, decreased compound muscle action potential, and prolonged latency of F-wave, is considered to be highly sensitive for early diagnosis of DSPN [16,49], but NSC cannot be used to assess therapeutic effects in diabetic patients [49]. Electromyography can be useful for detecting abnormal spontaneous activities in distal muscles in moderate to severe DSPN [50], although this test is also useful for ruling out other neuropathies, such as radiculopathies, mononeuropathies, or myopathies. In this study, we could not find axonal involvement without demyelination within DSPN patients. In T2DM, segmental demyelination is prominent with a milder axonal involvement whereas axonal loss is more severe in T1DM [51,52]. Initially, we considered abnormal electromyographic findings with abnormal NCS (mixed type) to be advanced or severe type DSPN, and diabetic patients with mixed type DSPN might show abnormal clinical and laboratory findings more frequently than those with demyelinated type DSPN. However, ML analysis and logistic regression did not effectively suggest any difference between the demyelinated and mixed types. Therefore, electrophysiological analysis is necessary to differentiate these two types of diabetic patients.

Numerous ML algorithms have been used to predict DM and diabetic complications such as retinopathy, nephropathy, foot ulceration and DSPN [24,25,26,27,28,29]. XGB is a scalable end-to-end tree boosting system [32] and is more suitable for small sample sizes unless the data are not highly dispersed when predicting glucose variability in T2DM patients [53]. SVM was used for microarray or high-dimensional data and is suitable for predicting DSPN in DM patients with a clinical data-based classification [24] and distinguishing retinopathy between diabetic patients and normal controls [26]. RF is an ensemble of decision trees and can minimize the individual error of trees [23]. RF has shown good performance in predicting the development and classification of DSPN based on clinical symptoms and examinations of type 1 diabetic patients [29]. Logistic regression analysis is a common statistical method used to develop a model for binary outcomes in the medical field [54] and can also be used as a supervised learning technique in ML methods. Even though various ML algorithms have been successfully developed as predictive models for the purpose of preventing the occurrence of diseases or their complications, some recent studies have shown that logistic regression has similar results to ML analysis [55,56], and attempts to combine logistic regression and ML methods also appear to enhance the performance of statistical methods in an automated manner [57]. In our study, the AUC of RF was superior to that of logistic regression when subjects were classified into two groups: confirmed vs. other combinations (Table 4), but the AUC of logistic regression was higher than that of ML algorithms for comparison between the demyelinated and mixed subgroups within the confirmed group (Table 6). The development of proper hybrid models for statistical and ML algorithms might increase the power of DSPN prediction in future studies.

In previous studies, numerous predisposing factors have been associated with DSPN in diabetic patients, particularly, duration of diabetes and HbA1c in T2DM patients [21,58]; moreover, old age, increased height, obesity, higher body mass index, poor glucose control, alcohol abuse, smoking, hypertension, cardiovascular disease, low level of HDL, dyslipidemia, hypertriglyceridemia, and microalbuminuria have also been shown to be risk factors in previous studies [18,19,20,21,58,59,60,61]. We found that the average values of numerous laboratory datapoints during the follow-up period (serum glucose, IFCC, HbA1c, albumin, and differential counts of lymphocytes and neutrophils) were important predisposing factors, as were clinical data such as height and disease duration (Table 5). The albumin has important antioxidant and anti-inflammatory properties, and the lower level of serum albumin was associated with the prevalence of DSPN or peripheral nerve dysfunctions in T2DM patients in previous studies [62,63] In our study, average value of HbA1c is the most sensitive node of a decision tree among the influence features, and average differential counts of lymphocytes and neutrophils are the second node (Figure 4c). Although there is no standardized decision-making algorithm for DSPN diagnosis, HbA1c qualifies as an important diagnostic criterion for DPSN because HbA1c a major risk factor for microvascular complications and closely associated with DSPN in T2DM [64] The neutrophil-lymphocyte ratio is an inflammatory marker and an important factor that predicts cardiovascular disease [65] and foot ulcer infection [66] in diabetic patients. Neutrophil level was also the most sensitive node for decision making of DPSN prediction in a previous study [67], and higher neutrophil-lymphocyte ratio might be related to chronic inflammatory process and increase the risk of DSPN [68].

In this study, we analyzed a small-sized sample, especially the probable group (*n* = 13), which might cause problems for pattern recognition and poor accuracy [69]. Many studies in the medical field often have only a small number of patients. In this study, we tried to increase the accuracy by dividing the patients into ten groups for use as a test set and a tenfold stratified cross validation set to compensate for the small sample size [31], but a more accurate prediction might be achieved with a larger number of diabetic patients. We further plan to perform ML analysis to predict various complications in diabetic patients in a prospective multicenter study and develop an application attached to an existing electronic health record system for easier transfer of patient data that can assist in predicting complications in diabetic patients. In addition, it was difficult to use deep learning model because insufficient sample size can lead to overfitting. If sufficient data is accumulated, it is possible to build deep learning model using time-series laboratory data or to apply a method of transfer learning with DSPN patient using pre-trained models for all diabetic patients.

## 5. Conclusions

In this study, we revealed that the ML algorithms, whose AUC values were superior to logistic regression, can be applied to type 2 DM patients to predict DSPN and that the classification depending only on clinical symptoms and signs of suspected DSPN was not appropriate for the application of ML algorithms to develop prediction models. In addition, ML algorithms cannot predict the type of electrophysiological features in DSPN, namely, demyelinated and mixed subgroups. We concluded that ML techniques, especially RF, can predict DSPN effectively when comparing the combination of the normal, possible, and probable groups with the confirmed group of DM patients and that electrophysiological analysis is important for identifying DSPN.

## Figures and Tables

**Table 1:** Identification of the selection of data and methods for machine learning analysis of subjects.

Feature Set Used	Lab Feature Extraction Method	Feature Counts	AUC	Accuracy (%)
Laboratory data only	Method 1	39	0.7954	73.74
	Method 2	39	0.7790	71.53
	Method 3	36	0.7226	65.32
	Method 1 + 3	75	0.8095	73.83
	Method 2 + 3	75	0.7950	72.26
	Method 1 + 2 + 3	114	0.8012	73.06
Clinical data only	-	30	0.7493	69.79
Laboratory and clinical data	Method 1	69	0.8284	76.09
	Method 2	69	0.8096	72.68
	Method 3	66	0.8100	72.98
	Method 1 + 3	105	0.8350	74.85
	Method 2 + 3	105	0.8141	73.02
	Method 1 + 2 + 3	144	0.8219	74.21

Note: method 1 = average value of each laboratory code during the follow-up period; method 2 = the first value of each laboratory code when T2DM was diagnosed initially; method 3 = the pattern of laboratory code changes (−1, 0, or 1), Abbreviations: AUC = area under the curve.

**Table 2:** Baseline characteristics of participants.

	Normal (A) (*n* = 93)	Possible (B) (*n* = 91)	Probable (C) (*n* = 13)	Confirmed (D) (*n* = 273)	*p*-Value	Post Hoc
Disease duration (days)	4543.18 ± 2849.75	4464.03 ± 2934.87	4933.46 ± 3463.31	5686.67 ± 3648.57	0.004	A<>D, B<>D
Age (years)	51.33 ± 12.30	49.74 ± 11.51	53.85 ± 8.92	51.32 ± 14.91	0.676	
Sex (male)	48 (51.6)	48 (52.7)	5 (38.5)	176 (64.5)	0.027	
Height (m)	1.61 ± 0.09	1.62 ± 0.09	1.59 ± 0.09	1.64 ± 0.09	0.006	A<>D
Weight (kg)	66.10 ± 11.76	66.26 ± 11.14	62.21 ± 11.41	64.29 ± 11.92	0.308	
BMI (kg/m^2^)	25.33 ± 3.81	25.26 ± 3.48	24.65 ± 4.07	23.82 ± 3.66	0.001	A<>D, B<>D
Initial BST	211.78 ± 98.75	196.51 ± 87.62	178.21 ± 104.57	249.06 ± 117.68	0.000	A<>D, B<>D
Initial HbA1c	8.69 ± 2.18	8.72 ± 2.06	9.03 ± 3.09	9.59 ± 2.54	0.002	A<>D, B<>D
DM retinopathy	25 (26.9)	26 (28.6)	3 (23.1)	141 (51.6)	0.000	
Hypertension	54 (58.1)	56 (61.5)	8 (61.5)	186 (68.1)	0.304	
Dyslipidemia	76 (81.7)	70 (76.9)	10 (76.9)	197 (72.2)	0.29	
Smoking						
No	61 (65.6)	57 (62.6)	12 (92.3)	163 (59.7)	0.172	
Current	18 (19.4)	19 (20.9)	1 (7.7)	57 (20.9)		
Past smoking	14 (15.1)	15 (16.5)	0 (0.0)	53 (19.4)		
Family history of DM	28 (30.1)	51 (56.0)	4 (30.8)	106 (38.8)	0.003	
CAD Hx	25 (26.9)	24 (26.4)	6 (46.2)	93 (34.1)	0.248	
CVD Hx	43 (46.2)	33 (36.3)	6 (46.2)	134 (49.1)	0.205	
Stroke Hx	25 (26.9)	16 (17.6)	2 (15.4)	63 (23.1)	0.427	
Diabetes education	43 (46.2)	36 (39.6)	8 (61.5)	128 (46.9)	0.412	
Medications						
Metformin	83 (89.2)	86 (94.5)	11 (84.6)	201 (73.6)	0.000	
Sulfonylureas	64 (68.8)	62 (68.1)	5 (38.5)	159 (58.2)	0.048	
TZDs	11 (11.8)	5 (5.5)	1 (7.7)	3 (13.6)	0.158	
DPP4is	62 (66.7)	65 (71.4)	8 (61.5)	147 (53.8)	0.011	
SGLT2is	16 (17.2)	19 (20.9)	1 (7.7)	21 (7.7)	0.004	
Insulin	37 (39.8)	32 (35.2)	8 (61.5)	179 (65.6)	0.000	
CCBs	32 (34.4)	26 (28.6)	7 (53.8)	104 (38.1)	0.204	
ACEis	10 (10.8)	10 (11.0)	1 (7.7)	32 (11.7)	0.965	
ARBs	51 (54.8)	54 (59.3)	7 (53.8)	156 (57.1)	0.933	
BBs	21 (22.6)	24 (26.4)	6 (46.2)	76 (27.8)	0.355	
Thiazides	15 (16.1)	20 (22.0)	2 (15.4)	47 (17.2)	0.723	
Statins	78 (83.9)	70 (76.9)	10 (76.9)	192 (70.3)	0.058	

Note: Values are presented as the mean ± standard deviation or number of subjects (%). *p* < 0.05 among the four groups by one-way ANOVA for continuous data or likelihood ratio for categorical data. Post hoc testing was performed using the Games–Howell test. Abbreviations: BMI = body mass index; BST = blood sugar test; HbA1c = hemoglobin A1c; DM = diabetes mellitus; Hx = history; CAD = coronary artery disease; CVD = cerebrovascular disease; TZDs = thiazolidinediones; DPP4is = dipeptidyl peptidase-4 inhibitors; SGLT2is = sodium-glucose cotransporter-2 inhibitors; CCBs = calcium channel blockers, ACEis = angiotensin-converting-enzyme inhibitors; ABRs = angiotensin II receptor blockers; BBs = beta blockers.

**Table 3:** Values of AUC and accuracy of machine learning analysis when comparing each group or their combinations.

Classification	ML Model Which Showed the Best Result	AUC	Accuracy (%)
A vs. B vs. C vs. D	XGB + RF	0.8546	60.85
A vs. B vs. C + D	RF	0.8105	62.34
A vs. B + C vs. D	RF	0.8075	61.32
A + B vs. C vs. D	XGB + RF	0.8925	73.40
A + B vs. C + D	RF	0.8103	72.68
A + B + C vs. D	RF	0.8250	74.47

Note: A = normal group, B = possible group, C = probable group, D = confirmed group. Abbreviations: AUC = area under the curve; XGB = XGBoost; RF = random forest; SVM = support vector machine.

**Table 4:** Values of machine learning and logistic regression analysis using the classification of the combination of the normal, possible, and probable groups versus the confirmed group.

Model	AUC	Accuracy (%)	Sensitivity	Specificity
XGB	0.7604	69.83	0.7708	0.5899
SVM	0.7535	66.81	0.6643	0.6721
RF	0.8250	74.47	0.7940	0.6720
XGB + SVM	0.7822	71.28	0.7712	0.6363
XGB + RF	0.8235	74.47	0.7927	0.6743
SVM + RF	0.8070	73.19	0.7957	0.6478
XGB + RF + SVM	0.8105	73.62	0.8103	0.6342
Logistic regression	0.6620	84.76	0.9721	0.3519

Abbreviations: AUC = area under the curve; XGB = XGBoost; RF = random forest; SVM = support vector machine.

**Table 5:** Top 69 influential features in the classification of the combination of the normal, possible, and probable groups versus the confirmed group.

Ranking	Feature Name	Importance Score	Ranking	Feature Name	Importance Score
1	Avg glucose	0.997768	36	Avg WBC	0.280162
2	Avg IFCC	0.794161	37	Avg PLT	0.262754
3	Avg HbA1c	0.789265	38	Avg chloride	0.250326
4	Avg albumin	0.731579	39	Avg uric acid	0.246706
5	Height	0.57069	40	CP IFCC	0.246499
6	Avg Diff count (lymphocyte %)	0.546759	41	CP creatinine (spot urine)	0.242497
7	Avg creatinine (spot urine)	0.493981	42	Avg MCV	0.240183
8	Avg Diff count (neutrophil %)	0.486409	43	Avg Diff count (eosinophil%)	0.237532
9	Disease duration	0.467576	44	Avg MCH	0.229848
10	Avg sodium	0.455435	45	Avg Diff count (monocyte %)	0.225926
11	Avg HCT	0.451166	46	CP HbA1c	0.225847
12	Avg ALT (GPT)	0.450865	47	Avg MCHC	0.222184
13	Avg RBC	0.417525	48	Avg bilirubin	0.217108
14	Avg Hb	0.383685	49	Avg free T4	0.208568
15	BMI	0.375055	50	CP urine SG	0.204239
16	Avg HDL	0.374211	51	Avg Diff count (basophil %)	0.201151
17	Avg BUN	0.351033	52	Diabetic retinopathy	0.176286
18	Avg AST (GOT)	0.348776	53	CP TG	0.155261
19	Avg ALP	0.342055	54	Use of insulin	0.14617
20	Avg BST	0.33438	55	CP HDL	0.146164
21	Avg creatinine	0.332449	56	CP cholesterol	0.127665
22	Age	0.319338	57	CP WBC	0.096003
23	Avg urine pH	0.31512	58	CP PLT	0.09567
24	Avg calcium	0.309396	59	Sex	0.084762
25	Avg TG	0.307935	60	CP BST	0.083089
26	Avg LDL	0.305571	61	CP ALP	0.080399
27	Avg TSH	0.303504	62	Smoking	0.068729
28	Avg protein	0.302998	63	CP creatinine	0.065407
29	CP glucose	0.297945	64	CP Diff count (lymphocyte %)	0.065285
30	CP urine pH	0.290718	65	CP bilirubin	0.060325
31	Avg cholesterol	0.287416	66	Use of sulfonylurea	0.05838
32	Avg potassium	0.286635	67	CP AST (GOT)	0.052956
33	Weight	0.285151	68	CP ALT (GPT)	0.050693
34	Avg urine SG	0.282845	69	Use of metformin	0.048544
35	CP LDL	0.280875			

Abbreviations: Avg = average; IFCC = International Federation of Clinical Chemistry; HbA1c = hemoglobin A1c; Diff = differential; HCT = hematocrit; ALT (GPT) = alanine aminotransferase (glutamic pyruvate transaminase); BST = blood sugar test; RBC = red blood cell; Hb = Hemoglobin; BMI= body mass index; HDL = high-density lipoprotein cholesterol; BUN = blood urea nitrogen; AST (GOT) = aspartate aminotransferase (glutamic oxaloacetic transaminase); ALP = alkaline phosphatase; TG = triglyceride; LDL = low-density lipoprotein cholesterol; TSH = thyroid-stimulating hormone; CP = change pattern; SG = specific gravity; WBC = white blood cell; PLT = platelet; MCV = mean cell volume; MCH = mean corpuscular hemoglobin; MCHC = mean corpuscular hemoglobin concentration; T4 = thyroxine.

**Table 6:** Machine learning and logistic regression results analyzing the demyelinated type vs. mixed type.

Model	AUC	Accuracy (%)	Sensitivity	Specificity
XGB	0.5492	62.39	0.8329	0.1797
SVM	0.5105	68.15	1.0000	0.0000
RF	0.5426	64.25	0.9245	0.0436
XGB + SVM	0.5698	67.78	0.9947	0.0000
XGB + RF	0.5579	64.52	0.9317	0.0378
SVM + RF	0.5457	67.41	0.9889	0.0000
XGB + RF + SVM	0.5601	67.41	0.9897	0.0000
Logistic regression	0.6350	70.97	0.8812	0.3889

Abbreviations: AUC = area under the curve; XGB = XGBoost; RF = random forest; SVM = support vector machine.

**Table 7:** Comparison of previous studies that used machine learning algorithms to predict DPSN in type 2 diabetes mellitus patients.

References	Criteria to Diagnose DSPN	Suggested ML Models	AUC/Accuracy	Laboratory Data Processing	Providing Decision-Making Tool
Kazemi et al., 2016 [24]	clinical (T1DM and T2DM)	MSVM	UC/0.76	UC	N
Dagliati et al., 2018 [25]	UC	LR	0.726/0.746	UC	nomogram
Fan et al., 2021 [27]	UC	EM	0.847/0.783	UC	N
Maeda-Gutierrez et al., 2021 [38]	clinical	RF	0.65/UC	UC	N
Current study	electrophysiological	RF	0.825/0.7447	average/change pattern	decision tree

Abbreviations: ML = machine learning, AUC = area under the curve; MSVM = multicategory support vector machine; LR = logistic regression; EM = ensemble model; RF = random forest; UC = uncheckable; N = none.

## Data Availability

The data presented in this study are available from the corresponding author upon reasonable request.

## References

[1] Center for Disease Control and Prevention (2020). National diabetes statistics report, 2020. Atlanta, GA: Centers for Disease Control and Prevention, US Department of Health and Human Services.

[2] Mohamadi A., Cooke D.W. (2010). Type 2 diabetes mellitus in children and adolescents. Adolesc. Med. State Art Rev..

[3] Russell J.W., Zilliox L.A. (2014). Diabetic neuropathies. Continuum.

[4] Feldman E.L., Callaghan B.C., Pop-Busui R., Zochodne D.W., Wright D.E., Bennett D.L., Bril V., Russell J.W., Viswanathan V. (2019). Diabetic neuropathy. Nat. Rev. Dis. Primers.

[5] Sloan G., Selvarajah D., Tesfaye S. (2021). Pathogenesis, diagnosis and clinical management of diabetic sensorimotor peripheral neuropathy. Nat. Rev. Endocrinol..

[6] Kaku M., Vinik A., Simpson D.M. (2015). Pathways in the diagnosis and management of diabetic polyneuropathy. Curr. Diabetes Rep..

[7] Tesfaye S., Boulton A.J., Dyck P.J., Freeman R., Horowitz M., Kempler P., Lauria G., Malik R.A., Spallone V., Vinik A. (2010). Diabetic neuropathies: Update on definitions, diagnostic criteria, estimation of severity, and treatments. Diabetes Care.

[8] Thomas P.K. (1997). Classification, differential diagnosis, and staging of diabetic peripheral neuropathy. Diabetes.

[9] Boulton A.J., Vinik A.I., Arezzo J.C., Bril V., Feldman E.L., Freeman R., Malik R.A., Maser R.E., Sosenko J.M., Ziegler D. (2005). Diabetic neuropathies: A statement by the American Diabetes Association. Diabetes Care.

[10] England J.D., Gronseth G.S., Franklin G., Miller R.G., Asbury A.K., Carter G.T., Cohen J.A., Fisher M.A., Howard J.F., Kinsella L.J. (2005). Distal symmetrical polyneuropathy: A definition for clinical research. A report of the American Academy of Neurology, the American Association of Electrodiagnostic Medicine, and the American Academy of Physical Medicine and Rehabilitation. Arch. Phys. Med. Rehabil..

[11] Dyck P.J., Kratz K.M., Karnes J.L., Litchy W.J., Klein R., Pach J.M., Wilson D.M., O’Brien P.C., Melton L.J. (1993). The prevalence by staged severity of various types of diabetic neuropathy, retinopathy, and nephropathy in a population-based cohort: The Rochester Diabetic Neuropathy Study. Neurology.

[12] Meijer J.W., Bosma E., Lefrandt J.D., Links T.P., Smit A.J., Stewart R.E., van der Hoeven J.H., Hoogenberg K. (2003). Clinical diagnosis of diabetic polyneuropathy with the diabetic neuropathy symptom and diabetic neuropathy examination scores. Diabetes Care.

[13] Himeno T., Kamiya H., Nakamura J. (2020). Lumos for the long trail: Strategies for clinical diagnosis and severity staging for diabetic polyneuropathy and future directions. J. Diabetes Investig..

[14] Bril V., Perkins B.A. (2002). Validation of the Toronto clinical scoring system for diabetic polyneuropathy. Diabetes Care.

[15] American Diabetes Association (2016). Standards of medical care in diabetes—2016 abridged for primary care providers. Clin. Diabetes A Publ. Am. Diabetes Assoc..

[16] England J.D., Gronseth G.S., Franklin G., Miller R.G., Asbury A.K., Carter G.T., Cohen J.A., Fisher M.A., Howard J.F., Kinsella L.J. (2005). Distal symmetric polyneuropathy: A definition for clinical research: Report of the American Academy of Neurology, the American Association of Electrodiagnostic Medicine, and the American Academy of Physical Medicine and Rehabilitation. Neurology.

[17] Pasnoor M., Dimachkie M.M., Kluding P., Barohn R.J. (2013). Diabetic neuropathy part 1: Overview and symmetric phenotypes. Neurol. Clin..

[18] Tesfaye S., Stevens L.K., Stephenson J.M., Fuller J.H., Plater M., Ionescu-Tirgoviste C., Nuber A., Pozza G., Ward J.D. (1996). Prevalence of diabetic peripheral neuropathy and its relation to glycaemic control and potential risk factors: The EURODIAB IDDM Complications Study. Diabetologia.

[19] Adler A.I., Boyko E.J., Ahroni J.H., Stensel V., Forsberg R.C., Smith D.G. (1997). Risk factors for diabetic peripheral sensory neuropathy. Results of the Seattle Prospective Diabetic Foot Study. Diabetes Care.

[20] Adler A. (2001). Risk factors for diabetic neuropathy and foot ulceration. Curr. Diabetes Rep..

[21] Tesfaye S., Chaturvedi N., Eaton S.E., Ward J.D., Manes C., Ionescu-Tirgoviste C., Witte D.R., Fuller J.H., EURODIAB Prospective Complications Study Group (2005). Vascular risk factors and diabetic neuropathy. N. Engl. J. Med..

[22] Bzdok D., Altman N., Krzywinski M. (2018). Statistics versus machine learning. Nat. Methods.

[23] Breiman L. (2001). Random forests. Mach. Learn..

[24] Kazemi M., Moghimbeigi A., Kiani J., Mahjub H., Faradmal J. (2016). Diabetic peripheral neuropathy class prediction by multicategory support vector machine model: A cross-sectional study. Epidemiol. Health.

[25] Dagliati A., Marini S., Sacchi L., Cogni G., Teliti M., Tibollo V., de Cata P., Chiovato L., Bellazzi R. (2018). Machine learning methods to predict diabetes complications. J. Diabetes Sci. Technol..

[26] Tsao H.Y., Chan P.Y., Su E.C.Y. (2018). Predicting diabetic retinopathy and identifying interpretable biomedical features using machine learning algorithms. BMC Bioinform..

[27] Fan Y., Long E., Cai L., Cao Q., Wu X., Tong R. (2021). Machine learning approaches to predict risks of diabetic complications and poor glycemic control in nonadherent type 2 diabetes. Front. Pharmacol..

[28] Schafer Z., Mathisen A., Svendsen K., Engberg S., Rolighed Thomsen T., Kirketerp-Moller K. (2020). Toward machine-learning-based decision support in diabetes care: A risk stratification study on diabetic foot ulcer and amputation. Front. Med..

[29] Haque F., Bin Ibne Reaz M., Chowdhury M.E.H., Srivastava G., Md Ali S.H., Bakar A.A.A., Bhuiyan M.A.S. (2021). Performance analysis of conventional machine learning algorithms for diabetic sensorimotor polyneuropathy severity classification. Diagnostics.

[30] American Diabetes Association (2020). Classification and diagnosis of diabetes: Standards of medical care in diabetes-2020. Diabetes Care.

[31] Kohavi R. (1995). A study of cross-validation and bootstrap for accuracy estimation and model selection. IJCAI.

[32] Chen T., Guestrin C. Xgboost: A scalable tree boosting system. Proceedings of the 22nd ACM SIGKDD International Conference on Knowledge Discovery and Data Mining.

[33] Hearst M.A., Dumais S.T., Osuna E., Platt J., Scholkopf B. (1998). Support vector machines. IEEE Intell. Syst. Appl..

[34] Sagi O., Rokach L. (2018). Ensemble learning: A survey. Wiley Interdiscip. Rev. Data Min. Knowl. Discov..

[35] Drummond C., Holte R.C. (2005). Severe class imbalance: Why better algorithms aren’t the answer. European Conference on Machine Learning.

[36] Ravi D., Wong C., Deligianni F., Berthelot M., Andreu-Perez J., Lo B., Yang G.Z. (2017). Deep learning for health informatics. IEEE J. Biomed. Health Inform..

[37] Kavakiotis I., Tsave O., Salifoglou A., Maglaveras N., Vlahavas I., Chouvarda I. (2017). Machine learning and data mining methods in diabetes research. Comput. Struct. Biotechnol. J..

[38] Maeda-Gutierrez V., Galvan-Tejada C.E., Cruz M., Valladares-Salgado A., Galvan-Tejada J.I., Gamboa-Rosales H., Garcia-Hernandez A., Luna-Garcia H., Gonzalez-Curiel I., Martinez-Acuna M. (2021). Distal symmetric polyneuropathy identification in type 2 diabetes subjects: A random forest approach. Healthcare.

[39] Meijer J.W., Smit A.J., Sonderen E.V., Groothoff J.W., Eisma W.H., Links T.P. (2002). Symptom scoring systems to diagnose distal polyneuropathy in diabetes: The diabetic neuropathy symptom score. Diabet. Med..

[40] Feldman E.L., Stevens M.J., Thomas P.K., Brown M.B., Canal N., Greene D.A. (1994). A practical two-step quantitative clinical and electrophysiological assessment for the diagnosis and staging of diabetic neuropathy. Diabetes Care.

[41] Perkins B.A., Olaleye D., Zinman B., Bril V. (2001). Simple screening tests for peripheral neuropathy in the diabetes clinic. Diabetes Care.

[42] Abraham A., Alabdali M., Alsulaiman A., Albulaihe H., Breiner A., Katzberg H.D., Aljaafari D., Lovblom L.E., Bril V. (2017). The sensitivity and specificity of the neurological examination in polyneuropathy patients with clinical and electrophysiological correlations. PLoS ONE.

[43] Franse L.V., Valk G.D., Dekker J.H., Heine R.J., van Eijk J.T. (2000). ‘Numbness of the feet’ is a poor indicator for polyneuropathy in Type 2 diabetic patients. Diabet. Med..

[44] Dyck P.J., Overland C.J., Low P.A., Litchy W.J., Davies J.L., Dyck P.J., O’Brien P.C., Albers J.W., Andersen H., Cl vs. NPhys Trial Investigators (2010). Signs and symptoms versus nerve conduction studies to diagnose diabetic sensorimotor polyneuropathy: Cl vs. NPhys. trial. Muscle Nerve.

[45] Chen X., Graham J., Dabbah M.A., Petropoulos I.N., Ponirakis G., Asghar O., Alam U., Marshall A., Fadavi H., Ferdousi M. (2015). Small nerve fiber quantification in the diagnosis of diabetic sensorimotor polyneuropathy: Comparing corneal confocal microscopy with intraepidermal nerve fiber density. Diabetes Care.

[46] Javed S., Petropoulos I.N., Tavakoli M., Malik R.A. (2014). Clinical and diagnostic features of small fiber damage in diabetic polyneuropathy. Handb. Clin. Neurol..

[47] Dyck P.J., Lais A., Karnes J.L., O’Brien P., Rizza R. (1986). Fiber loss is primary and multifocal in sural nerves in diabetic polyneuropathy. Ann. Neurol..

[48] Galiero R., Ricciardi D., Pafundi P.C., Todisco V., Tedeschi G., Cirillo G., Sasso F.C. (2021). Whole plantar nerve conduction study: A new tool for early diagnosis of peripheral diabetic neuropathy. Diabetes Res. Clin. Pract..

[49] Petropoulos I.N., Ponirakis G., Khan A., Almuhannadi H., Gad H., Malik R.A. (2018). Diagnosing diabetic neuropathy: Something old, something new. Diabetes Metab. J..

[50] Perkins B., Bril V. (2014). Electrophysiologic testing in diabetic neuropathy. Handb. Clin. Neurol..

[51] Sima A.A., Zhang W. (2014). Mechanisms of diabetic neuropathy: Axon dysfunction. Handb. Clin. Neurol..

[52] Sima A.A., Kamiya H. (2006). Diabetic neuropathy differs in type 1 and type 2 diabetes. Ann. N. Y. Acad. Sci..

[53] Elhadd T., Mall R., Bashir M., Palotti J., Fernandez-Luque L., Farooq F., Mohanadi D.A., Dabbous Z., Malik R.A., Abou-Samra A.B. (2020). Artificial Intelligence (AI) based machine learning models predict glucose variability and hypoglycaemia risk in patients with type 2 diabetes on a multiple drug regimen who fast during ramadan (The PROFAST—IT Ramadan study). Diabetes Res. Clin. Pract..

[54] Kleinbaum D.G., Klein M. (2010). Introduction to logistic regression. Logistic Regression.

[55] Panesar S.S., D’Souza R.N., Yeh F.C., Fernandez-Miranda J.C. (2019). Machine learning versus logistic regression methods for 2-year mortality prognostication in a small, heterogeneous glioma database. World Neurosurg. X.

[56] Lynam A.L., Dennis J.M., Owen K.R., Oram R.A., Jones A.G., Shields B.M., Ferrat L.A. (2020). Logistic regression has similar performance to optimised machine learning algorithms in a clinical setting: Application to the discrimination between type 1 and type 2 diabetes in young adults. Diagn. Progn. Res..

[57] Levy J.J., O’Malley A.J. (2020). Don’t dismiss logistic regression: The case for sensible extraction of interactions in the era of machine learning. BMC Med. Res. Methodol..

[58] Liu X., Xu Y., An M., Zeng Q. (2019). The risk factors for diabetic peripheral neuropathy: A meta-analysis. PLoS ONE.

[59] Andersen S.T., Witte D.R., Dalsgaard E.M., Andersen H., Nawroth P., Fleming T., Jensen T.M., Finnerup N.B., Jensen T.S., Lauritzen T. (2018). Risk factors for incident diabetic polyneuropathy in a cohort with screen-detected type 2 diabetes followed for 13 years: ADDITION-Denmark. Diabetes Care.

[60] Callaghan B.C., Gao L., Li Y., Zhou X., Reynolds E., Banerjee M., Pop-Busui R., Feldman E.L., Ji L. (2018). Diabetes and obesity are the main metabolic drivers of peripheral neuropathy. Ann. Clin. Transl. Neurol..

[61] Callaghan B.C., Price R.S., Feldman E.L. (2015). Distal symmetric polyneuropathy: A review. JAMA.

[62] Li L., Liu B., Lu J., Jiang L., Zhang Y., Shen Y., Wang C., Jia W. (2015). Serum albumin is associated with peripheral nerve function in patients with type 2 diabetes. Endocrine.

[63] Yan P., Tang Q., Wu Y., Wan Q., Zhang Z., Xu Y., Zhu J., Miao Y. (2021). Serum albumin was negatively associated with diabetic peripheral neuropathy in Chinese population: A cross-sectional study. Diabetol. Metab. Syndr..

[64] Su J.B., Zhao L.H., Zhang X.L., Cai H.L., Huang H.Y., Xu F., Chen T., Wang X.Q. (2018). HbA1c variability and diabetic peripheral neuropathy in type 2 diabetic patients. Cardiovasc. Diabetol..

[65] Azab B., Chainani V., Shah N., McGinn J.T. (2013). Neutrophil-lymphocyte ratio as a predictor of major adverse cardiac events among diabetic population: A 4-year follow-up study. Angiology.

[66] Altay F.A., Kuzi S., Altay M., Ates I., Gurbuz Y., Tutuncu E.E., Senturk G.C., Altin N., Sencan I. (2019). Predicting diabetic foot ulcer infection using the neutrophil-to-lymphocyte ratio: A prospective study. J. Wound Care.

[67] Metsker O., Magoev K., Yakovlev A., Yanishevskiy S., Kopanitsa G., Kovalchuk S., Krzhizhanovskaya V.V. (2020). Identification of risk factors for patients with diabetes: Diabetic polyneuropathy case study. BMC Med. Inform. Decis. Mak..

[68] Liu S., Zheng H., Zhu X., Mao F., Zhang S., Shi H., Li Y., Lu B. (2017). Neutrophil-to-lymphocyte ratio is associated with diabetic peripheral neuropathy in type 2 diabetes patients. Diabetes Res. Clin. Pract..

[69] Combrisson E., Jerbi K. (2015). Exceeding chance level by chance: The caveat of theoretical chance levels in brain signal classification and statistical assessment of decoding accuracy. J. Neurosci. Methods.

